# Dentists´ knowledge about common risk factors for cardiovascular diseases and periodontitis: An opportunity to be part of a multidisciplinary team

**DOI:** 10.4317/jced.60766

**Published:** 2024-05-01

**Authors:** Johanna Otero, Yamileth Ortiz-Gomez, Patricio Lopez-Jaramillo

**Affiliations:** 1 DDS, MPH. Faculty of Dentistry, Universidad Santo Tomás, Bucaramanga, Colombia. MASIRA Research Institute, Universidad de Santander, Bucaramanga, Colombia; 2 PhD. Doctoral Program in Public Health, Universidad El Bosque, Bogotá, Colombia; 3 MD, PhD. MASIRA Research Institute, Universidad de Santander, Bucaramanga, Colombia. Universidad UTE, Facultad de Ciencias de la Salud Eugenio Espejo, Quito, Ecuador

## Abstract

**Background:**

Reducing the disease burden of cardiovascular diseases and periodontitis continues to be a priority and dentists are part of the primary care team. However, it is unclear whether Colombian dentists have the necessary knowledge to identify cardiovascular disease risk factors in clinical practice. The main aim of this study was to investigate Colombian dentists’ knowledge about common risk factors for cardiovascular disease and periodontitis.

**Material and Methods:**

A cross-sectional study was conducted. A self-administered electronic survey was validated by experts and completed by 232 dentists who practiced in Colombia. Descriptive and multivariate analyzes were performed, including hierarchical cluster analysis.

**Results:**

Regarding the identification of shared risk factors for the development of cardiovascular disease or periodontitis, 80.6% identified smoking and 72.8% diabetes. The correct identification of cardiovascular disease risk factors was between 34.9% in the case of eating practices and 78.0% for physical inactivity. Being a woman (79.8%), under 40 years of age (64.0%), not having completed a postgraduate degree (68.5%), and working in private institutions (88.8%) were the characteristics of dentists that best identified common risk factors for both cardiovascular disease and periodontitis.

**Conclusions:**

Colombian dentists had good knowledge of common risk factors for both cardiovascular diseases and periodontitis, but limited knowledge about cardiovascular disease risk factors. Younger dentists better identified risk factors. Conversely, older, more experience, and more specialized practitioners could benefit from more training about cardiovascular risk factors in order to truly be part of multidisciplinary teams in primary care.

** Key words:**Dentists, Cardiovascular Diseases, Periodontitis, Health Knowledge, Attitudes, Practice, Patient Care Team.

## Introduction

Cardiovascular disease (CVD) is the leading cause of death in the world, particularly in low- and middle-income countries, where 80% of cases occur. It has been documented that more than 70% of CVDs can be attributed to modifiable risk factors, with smoking, cholesterol, and hypertension being the major risk factors (1). Oral and cardiovascular diseases are related chronic conditions, highly prevalent, with serious economic and health consequences, which greatly reduce the quality of life of those affected (2-5).

The evidence is clear that CVD and periodontitis are multifactorial conditions that share risk factors such as smoking, age, and diabetes, and have a common inflammatory pathway (4,6,7). Additionally, periodontitis is a chronic inflammatory disease that could increase the risk of developing CVD, particularly if it is severe or of endodontic origin, due to increased systemic inflammation (6,8,9). Therefore, it has been proposed that interventions aimed to reduce oral inflammation could have a role in the prevention of CVD and its complications (10). However, studies that have evaluated dental practice suggest that dentists are not evaluating the proposed relationship between oral and cardiovascular health (11), and that there is a lack of knowledge about the proper management of patients with hypertension (12). Nonetheless, dentists have reported feeling confident about treating patients with CVD or diabetes and agree that they should be trained to identify risk factors for oral-systemic disease (13).

Colombia is going through a reform of the health system, and this is a timely moment to help inform decision makers about the potential role of dentists in multidisciplinary teams in the primary prevention of CVD. To this end, the main objective of the present study was to investigate Colombian dentists’ knowledge about common risk factors for periodontitis and cardiovascular. The secondary objective was to identify groups of dentists who were best able to identify the risk factors.

## Material and Methods

A cross-sectional study of practicing dentists in Colombia was conducted between September and October 2022. This study was approved by the Ethics Committee of the University of Santander and all participants provided consent.

-Setting and data collection

Participants were recruited from three anonymized databases that included a total of 1,442 dental practitioners: graduates of Santo Tomas University in Bucaramanga; members of the Biomep dental society in Bucaramanga; and members of the Colombian Dental Federation. Sample size was calculated using Epidat version 4.1 software assuming that at least 75% of dentists would correctly identify risk factor for cardiovascular disease (13), a confidence level of 95%, and a precision of 5%. With these parameters, the expected sample was 241 dentists. Simple random sampling was carried out to select the sample, however, after three weeks of sending the invitation via email, the response was poor (n= 67), so we decided to use the snowball sampling method and seek volunteers via social networks or by referral from those who had already responded. Hypothesis test of equality of proportions (Chi-square test) and means (two-sample t test) of the characteristics of the participants selected by simple random sampling and those who answered the survey by snowball sampling were tested, and no significant differences were found between the samples (*p*>0.05).

The survey was designed based on the questions applied in the studies by Wilder, *et al*. 2014 (11) and Paquette, *et al*. 2015 (13). The validity of the instrument was evaluated with the participation of 10 thematic experts considered stakeholders from clinical practice, scientific societies, and territorial entities. The experts had postgraduate degrees in periodontics, endodontics, microbiology and research, all with at least 10 years of experience, and eight were university professors. The validity estimate was assessed using Lawshe Content Validity Ratio (0.83) and internal consistency was assessed using the Cronbach Alpha (0.77). The first part of the survey included sociodemographic variables such as sex, age and educational level, variables related to dental practice such as hours of care practice, type of practice (individual or group -shared with other colleagues or professionals-), type of institution (private or public), and availability of support staff. The second part of the survey related to the knowledge component, and included variables related to continuing professional development, knowledge of cardiovascular risk programs and identification of risk factors.

-Statistical analyses

Data are expressed as means (SD) for continuous variables and frequency (%) for categorical variables. The Chi-square test or Fisher exact test along with binary comparisons was used to analyze categorical variables, and Kruskal-Wallis test was used to analyze continuous variables. Additional post hoc comparisons using Bonferroni were performed to test significant differences between individual groups. A multivariate analysis that used sociodemographic and knowledge variables through 2-step agglomerative hierarchical cluster analysis was performed using the average linkage distance for grouping similar cases together in node. The final optimal number of clusters was then determined by Calinski–Harabasz procedures. All analyses were performed using Stata Statistical Software (17.0 BE-Basic Edition; StataCorp, Texas, USA).

## Results

Participants’ characteristics are shown in  Table 1. A response was obtained from 232 dentists who had at least 4 hours of practice per day (7.3 ± 1.9 hours). The mean age was 39.8 ± 12.0 years, 69.0% were women, 38.8% had achieved specialization or master’s degree training in clinical areas of dentistry, 73.3% exercised their practice individually, the most linked to private institutions (86.6%) and 74.2% had an assistant or hygienist in their work team.

 Table 2 shows participants’ knowledge of risk factors for oral and cardiovascular disease. In general, 65.9% of the dentists reported that they kept their knowledge up to date, 63.4% knew of a cardiovascular risk program. One in two dentists considered that poor oral hygiene (53.0%), diet (50.0%) and alcohol intake (48.3%) were shared risk factors for the development of these pathologies. Dyslipidemias were the least recognized as a risk factor (24.1%), while tobacco (80.6%) and diabetes (72.8%) were the most recognized. The identification as risk factors for the development of CVD of physical inactivity were (78.0%), hypertension (57.8%) and obesity (59.5%).

 Table 3 shows the results of the cluster analysis. The largest number of dentists was classified in Cluster 1 (45.7%). The dentists that best identify the risk factors for developing periodontitis, CVD, or both were found in Cluster 2 (38.6% of the participants), which characteristic were being a woman (79.8%), being less than 40 years (64.0%), a low proportion of people who have reached the level of specialty training or clinical master’s degree (31.5%) and group practice (23.6%), work in private institutions (88.8%), dedicate 7.3 ± 1.6 hours of care, having up-to-date knowledge (69.7%), knowing a cardiovascular risk program (59.5%) and a high proportion of dentist respondents were willing to participate in a multidisciplinary team.

## Discussion

The main objective of the present study was to investigate Colombian dentists’ knowledge about common risk factors for both CVD and periodontitis. The results suggest that a high proportion of dentists in Colombia can identify common risk factors for the development of CVD and periodontitis, such as smoking and diabetes. The secondary objective was to investigate the characteristics of dentists who best identified risk factors, and we found that younger dentists with no clinical specialty had greater knowledge. Interestingly, two thirds of dentists reported that they keep their knowledge up to date, more than a half were aware of a cardiovascular risk program and the vast majority were willing to participate in a multidisciplinary team, a situation that could favor the identification of risk factors in their clinical practice to better management of CVD and common CVD risk factors, and participation in multidisciplinary teams in primary care.

In contrast to our findings, Paquette and colleagues (13) found high awareness of risk factors for cardiovascular diseases such as diet, smoking, physical inactivity, obesity, and dyslipidemia. Our study, only 36.2% of dentists were aware that alcohol consumption was a major risk factor for CVD, 34.9% poor diet, and it is striking that the risk factor in which had the most doubts is dyslipidemia (24.1%). And similarly, Paquette and colleagues (13) found that a high proportion of dentists consider factors such as alcohol consumption, poor diet, high blood pressure and poor oral hygiene to be shared risk factors for the development of periodontitis and cardiovascular disease.

The relationship between periodontal disease and diabetes, and cardiovascular disease was recognized by most dentists, this finding is similar to other studies (13,14) and is complemented by dentists are aware of the association between oral health and systemic health. These findings are noteworthy since, when reviewing the knowledge for the management of patients with heart disease, dentists perceive that they have difficulties in managing this type of patient (15). Encouragingly, studies suggest that dentists are willing to improve their knowledge through continuing education programs (13,15).

Complementarily, dentists have also expressed willingness to help screen for cardiovascular disease risk factors, such as obesity and high blood pressure. Therefore, it is considered that dentists who include such practices may share an integral perspective regarding patient health and view their roles as health care professionals extending beyond the oral cavity (16). One might expect that knowledge of CVD and periodontitis risk factors would be greater in more experienced practitioners, this could be favorable their incorporation into the activities of multidisciplinary primary health care teams in charge of controlling cardiovascular risk factors (11). However, our findings suggest that the dentists with the best knowledge of cardiovascular risk factors are the youngest, that is, with less clinical experience.

In this regard, governing bodies and other decision makers have been encouraged to consider the incorporation of CVD risk factor assessments in dental care settings, considering that dentists must have the relevant skills from undergraduate to perform medical examinations and identify risk, decision makers believe that clinical experience can influence in practice, but that dentists’ knowledge must be strengthened using continuing education strategies (17). Complementarily, Junior dentists are more willing to assess blood pressure in practice, particularly if there are financial incentives (18).

In the present study, dentists with the greatest knowledge tended to work in individual practice, which may give them more time to assess the potential risks of cardiovascular disease in people with periodontitis (13). In favor of this, people who attend dental treatment can allow the assessment of risk factors for the development of systemic diseases and recognize that the dentist can participate in the prevention and early detection of diseases, and the prevention of complications of diseases other than those related to the oral cavity (19). The availability of patients who come to the dental office is an opportunity scenario to develop strategies such as the task shifting between physicians, nursing staff and dentists. This strategy is part of the HEARTS Initiative to improve awareness, treatment, and control of hypertension. HEARTS has been adopted by several countries in Latin America and the Caribbean, including Colombia, for the development of cardiovascular risk programs (20).

To reinforce, a call to action has been issued to low- and middle-income countries to promote the inclusion of oral health in all policies, highlighting the need for a bolder and more radical preventive approach that requires integrated and coordinated strategies with the agenda of the prevention of chronic non-communicable diseases (21). Collaborations between oral health and chronic disease programs may help address common risk factors for oral health and chronic disease (22) and increase the access to and the effectiveness of cardiovascular prevention programs (23). Our results in the dentists´ knowledge, even attitudes and practices, may be different from the findings of other studies that have been developed in countries that have health systems with different characteristics than ours, in addition to high income level, such as the United States (11,13,16), Sweden (17), Netherlands (18) and Saudi Arabia (12,15,19).

Finally, in this scenario of opportunity, since the physician who are usually in charge of cardiovascular risk programs recognize the link between oral and systemic diseases, which leads them to refer patients to the dentist and strengthen multidisciplinary networks that improve barriers interactions with dentists (24). The physicians, including clinical specialists, show interest in oral health, make evident the poor collaboration with dentists and highlight the need to promote collaborative practices (25). Colombia has detected the importance of the formation of multidisciplinary teams and recently a proposal from the Ministry of Health for the reform of the Colombian Health System gives special importance to the formation of multidisciplinary teams.

This study has strengths and limitations. We reviewed findings from five countries dentists´ knowledge about common risk factors for cardiovascular diseases and periodontitis, none from the Latin America & Caribbean Region, we consider this to be a strength to inform changes in the health policies of our Region. The cluster analyzes complement the description of knowledge, since they allow the identification of similarities between the answers of the participants and the establishment of a profile of dentists who best identify cardiovascular risk factors and those shared with periodontitis. The findings of the present study may be used to improve dentists’ knowledge of the oral systemic disease links to enhance patient referral to physicians for positive cardiovascular health outcomes. However, the questionnaires were self-administered, the participants answered the questions autonomously, so the result should be interpreted with caution and not generalized. Respondents who were recruited by snowball sampling were generally those who had an interest in the topic, so they may have given biased responses in the survey.

## Conclusions

In our study, dentists in Colombia have some knowledge about major risk factors for both cardiovascular diseases and periodontitis. However, there are some gaps in the discernment of risk factors that are not common or only have evidence for the development of cardiovascular disease, such as dietary practices, hypertension, dyslipidemia, and obesity. With greater knowledge in these aspects, the younger dentists who do not yet have postgraduate studies stand out, we suggest promoting the participation of this group of dentists in the cardiovascular risk programs of primary care. It is necessary to strengthen the knowledge of older dentists, with more experience or who have a postgraduate degree. Implementing continuing education strategies to improve knowledge and promoting collaborative practices in the primary care will improve the participation of dentists in multidisciplinary teams for the prevention and management of cardiovascular diseases.

## Figures and Tables

**Table 1:** Participants’ characteristics.

Characteristics	Female(n= 160)	Male(n= 72)	Total(n= 232)
Age -mean (SD)	39.4 (11.7)	40.9 (12.7)	39.8 (12.0)
Hours of care practice -mean (SD)	7.2 (1.9)	7.4 (1.9)	7.3 (1.9)
Education -n (%)			
Professional degree	64 (40.0)	26 (36.1)	90 (38.8)
Clinical specialty	57 (35.6)	33 (45.8)	90 (38.8)
Other specialty	32 (20.0)	9 (12.5)	41 (17.8)
Master	7 (4.4)	4 (5.6)	11 (4.7)
Practice type -n (%)			
Group	42 (26.2)	20 (27.8)	62 (26.7)
Individual	118 (73.7)	52 (72.2)	170 (73.3)
Institution type -n (%)			
Public	25 (15.6)	6 (8.3)	31 (13.4)
Private	135 (84.4)	66 (91.7)	201 (86.6)
Support staff -n (%)			
Assistant or dental hygienist	114 (71.2)	58 (80.6)	172 (74.1)
No support	46 (28.7)	14 (19.4)	60 (25.9)

**Table 2:** Proportion of knowledge of risk factors for the development of periodontitis and CVD.

Risk factor	Periodontitis not CVD	CVD not periodontitis	Periodontitis & CVD	Not know
Tobacco use -n (%)	31 (13.4)	9 (3.9)	187 (80.6)	5 (2.2)
Alcohol use -n (%)	13 (5.6)	84 (36.2)	112 (48.3)	23 (9.9)
Diet -n (%)	24 (10.3)	81 (34.9)	116 (50.0)	11 (4.7)
Physical inactivity -n (%)	8 (3.4)	181 (78.0)	33 (14.2)	10 (4.3)
Poor oral hygiene -n (%)	103 (44.4)	5 (2.2)	123 (53.0)	1 (0.4)
Hypertension -n (%)	7 (3.0)	134 (57.8)	85 (36.6)	6 (2.6)
Diabetes -n (%)	40 (17.2)	17 (7.3)	169 (72.8)	6 (2.6)
Dyslipidemias -n (%)	8 (3.4)	104 (44.8)	64 (27.6)	56 (24.1)
Obesity -n (%)	7 (3.0)	138 (59.5)	75 (32.3)	12 (5.2)

**Table 3:** Comparison of sociodemographic and knowledge characteristics in dentist’s clusters.

Characteristics	Cluster 1 (n= 106)	Cluster 2 (n= 89)	Cluster 3 (n= 37)	p-Value
Sex, Female -n (%)	61 (57.5)	71 (79.8)	28 (75.7)	0.002
Age, >=40 years old -n (%)	59 (55.7)	32 (36.0)	18 (48.6)	0.022
Education, Clinical specialty -n (%)	55 (51.9)	28 (31.5)	7 (18.9)	<0.001
Practice type, Group -n (%)	34 (32.1)	21 (23.6)	7 (18.9)	0.207
Institution type, Private -n (%)	95 (89.6)	79 (88.8)	27 (73.0)	0.028
Hours of care practice -mean (SD)	7.25 (2.1)	7.27 (1.6)	7.62 (1.9)	0.4772
Updated knowledge -n (%)	65 (61.3)	62 (69.7)	26 (70.3)	0.393
Meet a cardiovascular risk program -n (%)	71 (67.0)	53 (59.5)	23 (62.2)	0.555
Willingness to participate in a multidisciplinary team -n (%)	97 (91.5)	80 (89.9)	30 (81.1)	0.205
Identifies tobacco use as a risk factor for periodontitis and CVD -n (%)	104 (98.1)	67 (75.3)	16 (43.2)	<0.001
Identifies alcohol use as a risk factor for periodontitis and CVD -n (%)	17 (16.0)	65 (73.0)	2 (5.4)	<0.001
Identifies diet as a risk factor for CVD -n (%)	28 (26.4)	47 (52.8)	6 (16.2)	<0.001
Identifies physical inactivity as a risk factor for CVD -n (%)	70 (66.0)	88 (98.9)	23 (62.2)	<0.001
Identifies poor oral hygiene as a risk factor for CVD -n (%)	23 (21.7)	51 (57.3)	29 (78.4)	<0.001
Identifies hypertension as a risk factor for CVD -n (%)	42 (39.6)	67 (75.3)	25 (67.6)	<0.001
Identifies diabetes as a risk factor for periodontitis and CVD -n (%)	97 (91.5)	60 (67.4)	12 (32.4)	<0.001
Identifies dyslipidemias as a risk factor for CVD -n (%)	38 (35.8)	66 (74.2)	0 (0.0)	<0.001
Identifies obesity as a risk factor for CVD -n (%)	47 (44.3)	73 (82.0)	18 (48.6)	<0.001
Identifies behavioral risk factors for periodontitis or CVD -n (%)	3 (2.8)	10 (11.2)	0 (0.0)	0.014
Identifies biological risk factors for periodontitis or CVD -n (%)	11 (10.4)	34 (38.2)	0 (0.0)	<0.001

## Data Availability

The data sets used and/or analyzed during the current study are available at https://github.com/danieladyro/knowledge_of_risk_factors.git Authors’ contributions: JO conceptualization, methodology, analysis, validation, writing-original manuscript, and review drafting. YO-G validation, review, and editing. YO-G and PL-J review and editing. All authors have read and agreed to the published version of the manuscript.
